# Predictive hybrid model of a grid-connected photovoltaic system with DC-DC converters under extreme altitude conditions at 3800 meters above sea level

**DOI:** 10.1371/journal.pone.0324047

**Published:** 2025-05-19

**Authors:** Jose Cruz, Luis Baca, Norman Beltran, Julio Chura, Helarf Calsina, Vilma Sarmiento, Reynaldo Condori, Saul Huaquipaco, Víctor Yana-Mamani, Wilson Negrão Macêdo, Wilson Mamani

**Affiliations:** 1 Faculty of FIMEES, Universidad Nacional del Altiplano, Puno, Perú; 2 Faculty of Engineering, Universidad Nacional de Juliaca, Juliaca, Peru; 3 Faculty of Engineering, Universidad Nacional de Moquegua, Moquegua, Peru; 4 Group for the Study and Development of Energy Alternatives, Federal University of Pará, Belém, Brazil; 5 University of Alicante, Alicante, Spain; Agricultural Sciences and Natural Resources University of Khuzestan, IRAN, ISLAMIC REPUBLIC OF

## Abstract

This study aims to develop a predictive hybrid model for a grid-connected PV system with DC-DC optimizers, designed to operate in extreme altitude conditions at 3800 m above sea level. This approach seeks to address the “curse of dimensionality” by reducing model complexity and improving its accuracy by combining the recursive feature removal (RFE) method with advanced regularization techniques, such as Lasso, Ridge, and Bayesian Ridge. The research used a photovoltaic system composed of monocrystalline modules, DC-DC optimizers and a 3000 W inverter. The data obtained from the system were divided into training and test sets, where RFE identified the most relevant variables, eliminating the reactive power of AC. Subsequently, the three regularization models were trained with these selected variables and evaluated using metrics such as precision, mean absolute error, mean square error and coefficient of determination. The results showed that RFE - Bayesian Ridge obtained the highest accuracy (0.999935), followed by RFE - Ridge, while RFE - Lasso had a slightly lower performance and also obtained an exceptionally low MASE (0.0034 for Bayesian and Ridge, compared to 0.0065 for Lasso). All models complied with the necessary statistical validations, including linearity, error normality, absence of autocorrelation and homoscedasticity, which guaranteed their reliability. This hybrid approach proved effective in optimizing the predictive performance of PV systems under challenging conditions. Future work will explore the integration of these models with energy storage systems and smart control strategies to improve operational stability. In addition, the application of the hybrid model in extreme climates, such as desert or polar areas, will be investigated, as well as its extension through deep learning techniques to capture non-linear relationships and increase adaptability to abrupt climate variations.

## 1. Introduction

One of the problems with regression and classification is that the number of input variables is very large, which begins to interfere with learning rather than helping to be more accurate; this is called the “curse of dimensionality” [1,2]. To eliminate this effect, there are methods such as using thresholds of variance and feature selection, and how they interact with each other, but they do not take into account the performance of the overall model. Thus, recursive feature removal (RFE) reduces model complexity by removing features individually until the optimal number of features remains [3–5]. Variable elimination methods, such as RFE, must take into account two fundamental criteria. The first is when a variable has little or no information about the property under study. The second is when a pair of variables provides the same information about the problem, because they are highly correlated. In this case, the RFE does not exactly correct this second point because it tends to assign the same importance to all the variables in the group in the initial stages. Therefore, a hybrid method is presented, and this disadvantage is corrected using other Shrinkage regularization variable selection methods: Lasso, Ridge, and Bayesian Ridge. While these methods are critical for modeling, their applicability and performance can be significantly affected by the specific operating conditions of PV systems. In particular, there is a gap in research on how these methods address the unique challenges imposed by operations at extreme altitudes, where factors such as extreme temperature variations, higher UV radiation, and lower air density can influence component behavior and correlation between input variables.

Solar panel efficiency is enhanced through DC-DC converters, which regulate voltage fluctuations inherent in photovoltaic systems [6–8]. In the field of DC-DC converters applied to solar panels [9], the design and validation, both in simulation and in physical prototype, of a DC-DC amplifier designed to control the current of a set of LEDs is presented [10], he proposed an architecture that combines two DC-DC converters with a shared interface to generate two differentiated outputs. This design uses a single controlled switch applied to a renewable energy conversion system that connects the solar panel, battery, and home loads. In a regulated environment [11], it performed a loop-power hardware simulation to safely assess the performance and reliability of the “PowerCorner” device, which was created to provide power to microgrids, batteries, and photovoltaic panels. This device contains two simulation modules: in the direct current (DC) section, the photovoltaic power factory and storage system are simulated using a DC power amplifier, and in the alternating current (AC) section, the rural grid is simulated using an AC power amplifier [12]. proposed a system to maximize power in solar panels using a solar tracker that implements maximum power point tracking (MPPT) in a DC-DC converter. They used a prototype with single-axis motion controlled by an op-amp and a PIC18F4520 microcontroller, highlighting its applicability to solar array arrays. Similarly [13], it developed a hardware-in-the-loop (PHIL)-based testbed that included a photovoltaic emulator and a DC grid emulator. These systems, also based on PHIL, offer greater testing flexibility than standalone source emulators, integrating LC filters and advanced control algorithms to improve system bandwidth and robustness. On the other hand [14], he presented an innovative digital control method for a multi-output DC-DC converter using PID feedback and a neural network-based predictive controller. This approach improves the dynamic characteristics of the converter, achieving a 45% reduction in the output voltage underpulse and a 26% reduction in the reactor under pulse.

In the field of prediction and modeling [15], he designed a solar irradiance sensor based on multiple linear regression, using current and voltage data as inputs, and validated the model with an error of 3.876 [16]. They applied a linear regression model to assess the impact of renewable energy use on household energy costs and concluded that installing solar panels on buildings and homes could significantly reduce costs and mitigate environmental issues [17]. It also used a hybrid linear regression model and constrained Boltzmann machines to improve the forecast of short-term PV power generation using production data from GEFCom2014. In an innovative approach [18], he designed a method to predict photovoltaics using multipoint solar irradiance measurements and color-based image analysis, achieving a 58% reduction in errors compared to uncorrected models.

Regarding regression model improvement techniques [19], implemented a two-stage approach using Random Forest and Recursive Feature Elimination (RFE) for feature selection and deep neural networks for electricity load and price prediction, outperforming other models compared. Similarly [20], it stressed the importance of reliable solar radiation and energy forecasting to optimise the planning of solar plants by conducting a comprehensive review of existing methods [21]. carried out a systematic analysis of DC-DC converters coupled to inductors, and [22] developed a slider mode control algorithm to optimize battery charging in electric vehicles. In addition [23], it presented an SLG backhaul control strategy in MATLAB/Simulink for Global Maximum Power Point (GMPPT) tracking under harsh conditions, while [24] it designed a control algorithm for a bidirectional converter connected to DC microgrids. Finally [25], he proposed a hybrid approach based on improved random forests with removal of recursive features to classify partial discharge sources, achieving an accuracy of 98.8%.

To address this identified gap, this work presents a hybrid model that combines recursive feature removal (RFE) with Shinkrage regularization techniques (Lasso, Ridge and Bayesian Ridge), specifically designed to mitigate the challenges of high altitude in the power prediction of PV plants with DC-DC systems. The novelty of this approach lies in its adaptation to consider the complexities introduced by this environment, seeking to improve the accuracy of estimates under extreme conditions.

Considering the problems and the background reviewed, the contributions of this research are Implementation and evaluation of a photovoltaic plant with a DC-DC system; Implementation of hybrid models for power prediction in DC-DC plants and Validation of proposed hybrid models.

## 2. Methodology

### 2.1 System description

#### 2.1.1 Power system model.

**Fig 1:**
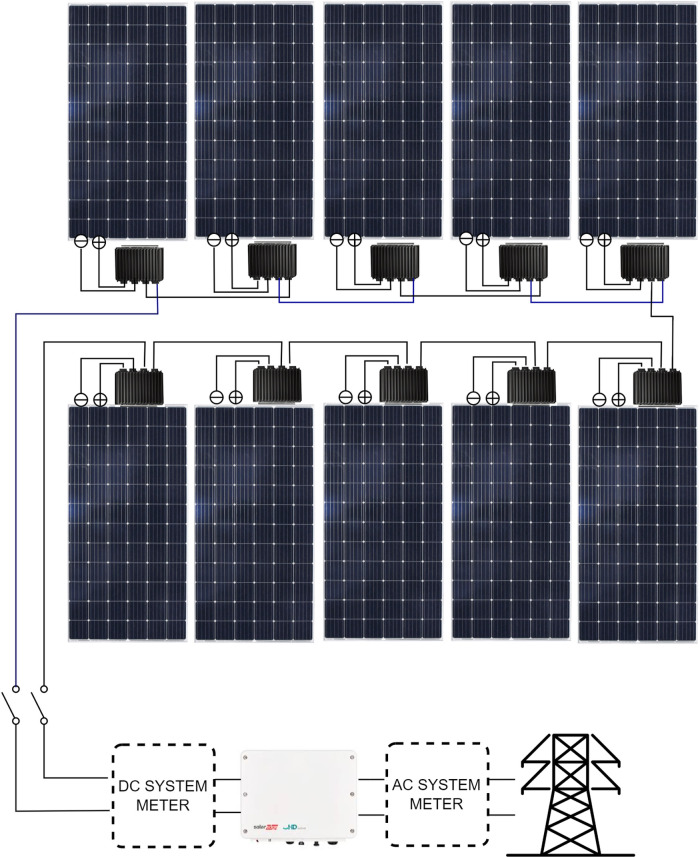
Diagram of GCFVS with DC-DC converters.

The grid-tied photovoltaic system (GCFVS) with DC-DC optimizers consists of ten 370 Wp monocrystalline photovoltaic modules of the ERA SOLAR ESPSC370 brand, ten Edge P370 DC-DC solar converters supporting up to 370 W of input power, and a single-phase inverter with HD-Wave Solar Edge SE3000H technology with an output power of 3000 W. No energy storage systems were installed. GCFVS has the configuration shown in Fig 1.

**Fig 2:**
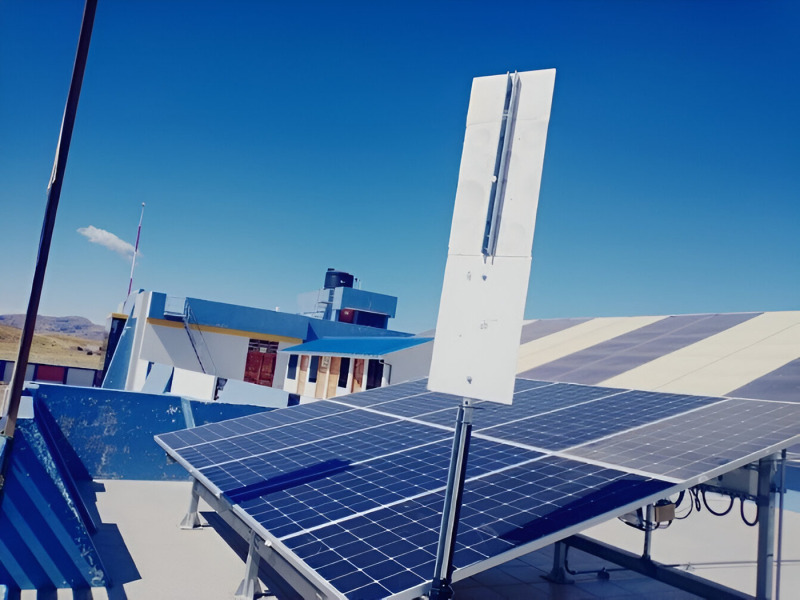
Shade and dust over SFCR.

The photovoltaic array is composed of ten panels, one of which has a layer of dust on its surface, while two others are partially shaded due to the presence of two poles, as illustrated in Fig 2.

#### 2.1.2 Data collection.

**Fig 3:**
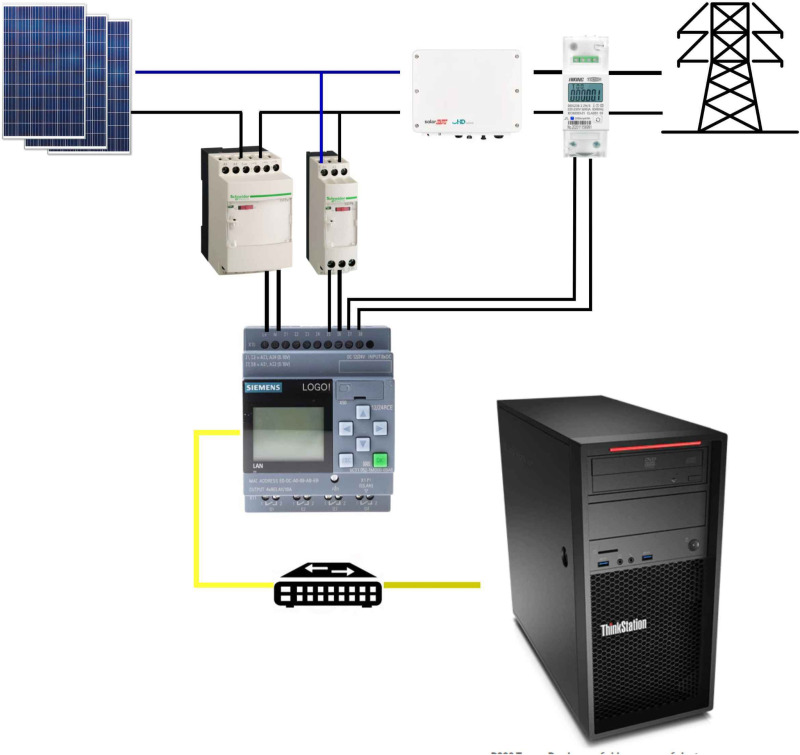
Data collection instrumentation diagram.

The instrumentation system for data acquisition used Schneider Zelio analog current and voltage transducers together with a HIKING TOMZN power meter that complies with the IEC 62053–21 standard. This device allows data logging using a micro-LOGO programmable logic controller (PLC) in its version 8.3, using the Modbus RS485 communication protocol and a Precision Class 1. The overall control of the system was managed using LABVIEW software, as illustrated in Fig 3. This design ensures efficient and accurate integration of components, facilitating real-time monitoring and analysis of electrical parameters.

The instruments were calibrated using FLUKE meters with calibration certificates following the guidelines set out in IEC 61724–1. A class A degree of control was assured, which included uncertainties in both types of current: alternating (AC) and direct (DC). In addition, the machinery used achieved an accuracy of 1% and the data was recorded with a sampling rate of 60 s, ensuring the reliability and accuracy of the measurements made.

### 2.2 Predictive models

**Fig 4:**
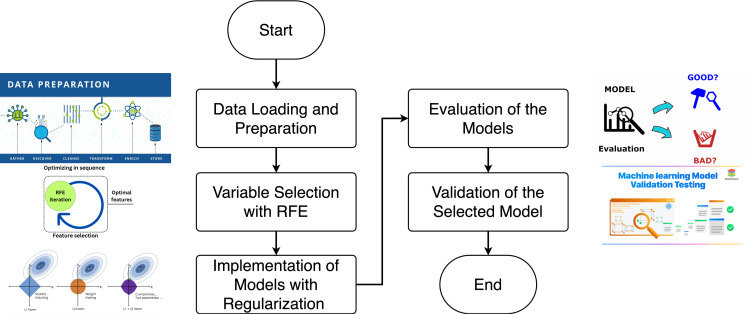
Flowchart.

Fig 4 shows the flowchart that describes the process of deploying and validating the models:

1. **Data Loading and Preparation**: The data corresponding to the CC plant were loaded and divided into two sets: a training set to train and adjust the proposed models, and a test set to evaluate the generalizability of the model. The segmentation was carried out through a cross-validation process, with the aim of guaranteeing adequate representativeness of the variables in both subsets. The statistical characteristics of the data are presented in Table 1

**Table 1:** Data set.

Variable	Count	Mean	Std	min	25%	50%	75%	Max
AC CURRENT	5041	7.40	4.87	0	2.83	7.03	12.98	13.84
AC VOLTAGE	5041	218.91	4.175	203.1	216.6	219.7	221.6	228.7
AC POWER	5041	1623.64	1095.59	0	600.1	1535.6	2872.8	3009
AC FRECUENCY	5041	59.99	0.039	59.86	59.96	59.99	60.02	60.21
AC APPARENT POWER	5041	1636.89	1086.65	0	614.0	1543.0	2877.6	3016
AC REACTIVE POWER	5041	149.48	41.955	0	135.07	154.10	176.31	216.71
DC CURRENT	5041	4.19	2.71	0	1.65	4.21	6.99	8.21
DC VOLTAGE	5041	368.47	78.69	0	370.0	370.1	370.3	445.7
DC POWER	5041	1648.21	1112.07	0	609.2	1558.4	2914.0	3055

1. **Variable selection with RFE:** RFE was applied to the training set to identify and select the most relevant variables. This method iteratively removes features that have the least impact on the model based on the relative importance of each variable. The result is a reduced set of variables that optimizes model performance and reduces computational complexity.
2. **Construction of models with regularization:** Three regression models with regularization were trained to control for overfitting and improve generalization using the variables selected by RFE: Lasso, which eliminates the predictors with less relevance; Ridge, which applies a regularization that penalizes the large coefficients and distributes the weight among all the selected variables; and Bayesian Ridge, which introduces a probabilistic approach that incorporates a priori distributions on the coefficients of the model.
3. **Model Evaluation:** All three models were evaluated using the test suite. The evaluation parameters used include accuracy, mean absolute error (MAE), mean square error (MSE), coefficient of determination, adjusted coefficient of determination, and training and testing times. The model that demonstrated the best performance among these indicators was chosen as the most appropriate.
4. **Validation of the selected model:** To ensure the statistical and practical validity of the chosen model, the following analyses were performed: linearity to confirm that there is a linear correlation between the predictor variables and the responding variable. Normality of error terms: Determine whether the residuals are governed by a normal distribution. Correlation and Autocorrelation: to check if the residuals maintain a correlation between them, which could indicate a model that has not been specified correctly. Homoscedasticity: Ensure that the variability of error terms remains constant across the entire range of projections.

#### 2.2.1 RFE.

The RFE method is a simple recursive process that classifies variables according to a measure of the importance of each variable given by a classifier. In each iteration, the relevance of all variables is measured and the least important is eliminated. In practice, to speed up the process, a group of variables is eliminated in each iteration, which is usually a small percentage of the total number of variables. Recursion in the ordering of variables improves performance when there are correlated variables [26].

The mathematical explanation of how RFE works is given below:

Step 1: Training the model: Initially, a model is trained with all the available features, where n is the number of features and $f(X;\theta )X=\left\{x_{1},x_{2},\cdots ,x_{n}\right\}$**θ** represents the parameters of the model. Depending on the model, an importance metric is calculated for each characteristic, **xi**. For example, in a linear model, this importance can be associated with weights **ωi**.

Step 2: Calculation of the combined importance: The importance r_i_ of a characteristic xi can be represented as a combination of two terms:


$$r_{i}=\beta \left|\omega_{i}\right|+(1-\beta )\frac{R_{i}}{Q_{S,i}}$$ (1)

Where:

ωi is the weight associated with the characteristic xi.

Ri is a metric of additional relevance, such as mean squared error or the impact on the target function by removing the xi feature.

QS,i: A normalization factor contingent on the subset S of current features, where S denotes the active feature space

β is an adjustment parameter (0 ≤ β ≤ 1) that controls the weighting between the magnitude of the weights and the relative relevance metric.

Step 3: Removing Features: Once the importance r i has been calculated for all features, the feature with the least importance is identified, i.e., the feature that minimizes r_i_:


$$x_{k}=arg\min_{i\in S}r_{i}$$ (2)

where S is the current subset of the features considered. This xk feature is removed from the S set, i.e., $S\leftarrow S\setminus \left\{x_{k}\right\}$

Step 4: Iteration: This process is repeated iteratively. At each step, the model f(X;θis retrained with the reduced set S, the importance ri is recalculated, and a feature is removed. This cycle continues until the desired number of previously defined final n-characteristics is reached.

The final S final set contains the most relevant final n characteristics that are optimally selected through the iterative process of removal and recalibration of importance.

#### 2.2.2 Ridge.

In a linear regression of the Ridge type, this is expressed by equation (3).


$$E=\sum_{i=1}^{N}{\left(y_{i}-{\hat{y}}_{i}\right)}^{2}$$ (3)

Like the dataset we use to make machine learning models, models must follow the Gaussian distribution defined by its mean μ and variance σ2 and is represented by N (μ, σ2), i.e., X∼N (μ, σ2), where X is the input matrix.

For any data point Xi, the probability density function is expressed as:


$$P\left(X_{i}\right)=\frac{1}{2\pi \sigma^{2}}e^{\frac{-1}{2}\frac{{\left(x_{i}-\mu \right)}^{2}}{\sigma^{2}}}$$ (4)

Each occurrence of Xi is independent of the occurrence of the others. The joint probability of each is given by


$$p\left(x_{1},x_{1},\ldots ,x_{N}\right)=\prod_{i=1}^{N}\frac{1}{2\pi \sigma^{2}}e^{\frac{-1}{2}\frac{{\left(x_{i}-\mu \right)}^{2}}{\sigma^{2}}}$$ (5)

The line containing the best fit for regression is shown in (6).


$$P\left(X|\mu \right)=p\left(x_{1},x_{1},\ldots ,x_{N}\right)=\prod_{i=1}^{N}\frac{1}{2\pi \sigma^{2}}e^{\frac{-1}{2}\frac{{\left(x_{i}-\mu \right)}^{2}}{\sigma^{2}}}$$ (6)

The probability function considers the natural logarithm to improve the bitline. Subsequently, it is equal to 0, as shown in (7).


$$ln(P\left(X|\mu \right))=ln(p\left(x_{1},x_{1},\ldots ,x_{N}\right))=$$ (7)

$$\ln\prod_{i=1}^{N}\frac{1}{2\pi \sigma^{2}}e^{\frac{-1}{2}\frac{{\left(x_{i}-\mu \right)}^{2}}{\sigma^{2}}}=\sum_{i=1}^{N}\ln\left(\frac{1}{2\pi \sigma^{2}}e^{\frac{-1}{2}\frac{{\left(x_{i}-\mu \right)}^{2}}{\sigma^{2}}}\right)$$ (8)

$$\sum_{i=1}^{N}\ln\left(\frac{1}{2\pi \sigma^{2}}\right)-\sum_{i=1}^{N}\frac{1}{2}\frac{{\left(x_{i}-\mu \right)}^{2}}{\sigma^{2}}$$ (9)

$$\frac{\partial ln\left(P\left(X|\mu \right)\right)}{\partial \mu }=\frac{\partial \sum_{i=1}^{N}\ln\left(\frac{1}{2\pi \sigma^{2}}\right)}{\partial \mu }-\frac{\partial \sum_{i=1}^{N}\frac{1}{2}\frac{{\left(x_{i}-\mu \right)}^{2}}{\sigma^{2}}}{\partial \mu }$$ (10)

$$=0+\sum_{i=1}^{N}\frac{\left(x_{i}-\mu \right)}{\sigma^{2}}=\sum_{i=1}^{N}\frac{\left(x_{i}-\mu \right)}{\sigma^{2}}$$ (11)

$$\frac{\partial ln\left(P\left(X|\mu \right)\right)}{\partial \mu }=\sum_{i=1}^{N}\frac{\left(x_{i}-\mu \right)}{\sigma^{2}}=0⟹\mu =\frac{\sum_{i=1}^{N}x_{i}}{N}$$ (12)

Whereas the probability (likelihood) L is equivalent to the error function E, as well as the Gaussian distribution with mean transposition (ω) * X and variability 2.


$$y\sim N\left(\omega^{T}X,\sigma^{2}\right)ory=\omega^{T}X+\varepsilon$$ (13)

When outliers are found, a normalization method is applied to the data to change the cost function and penalize high weights [27], as illustrated in (14).


$$I_{RIDGE}=\sum_{I=1}^{N}{\left(y_{i}-{\hat{y}}_{i}\right)}^{2}+\lambda |\omega {|}^{2}$$ (14)

$$|\omega {|}^{2}=\omega^{T}\omega =\omega_{1}^{2}+\omega_{2}^{2}+\cdots +\omega_{D}^{2}$$ (15)

There are two probabilities:


$$J=\left(Y-X_{\omega }\right){\left(Y-X_{\omega }\right)}^{T}+\lambda \omega^{T}\omega =Y^{T}T-2Y^{T}X\omega +\omega^{T}X^{T}X\omega +\lambda \omega^{T}\omega$$ (16)

Posterior


$$P\left(Y|X,\omega \right)=\prod_{i=1}^{N}\frac{1}{2\pi \sigma^{2}}\exp\left(-\frac{1}{2\sigma^{2}}{\left(y_{n}-\omega^{T}x_{n}\right)}^{2}\right)$$ (17)

A priori


$$AP(\omega )=\frac{\lambda }{\sqrt{2\pi }}\exp\left(-\frac{\lambda }{2}\omega^{T}\omega \right)$$ (18)

#### 2.2.3 Lasso.

In the same way for Lasso [28]


$$J_{LASSO}=\sum_{n=1}^{N}{\left(y_{i}-{\hat{y}}_{i}\right)}^{2}+\lambda \left\|\omega \right\|$$ (19)

Maximizing Probability


$$P\left(Y|X,\omega \right)=\prod_{i=1}^{N}\frac{1}{2\pi \sigma^{2}}\exp\left(-\frac{1}{2\sigma^{2}}{\left(y_{n}-\omega^{T}x_{n}\right)}^{2}\right)$$ (20)

A priori


$$P(\omega )=\frac{\lambda }{2}\exp\left(-\lambda |\omega |\right)$$ (21)

Then


$$J=\left(Y-X_{\omega }\right){\left(Y-X_{\omega }\right)}^{T}+\lambda |\omega |$$ (22)

And


$$\frac{\partial J}{\partial \omega }=-2X^{T}Y+2X^{T}Y+2X^{T}X_{\omega }+\lambda sign(\omega )=0$$ (23)

Whereas


sign(ω)=1,Ifx>0and−1ifx<0and0ifx=0 (24)

#### 2.2.4 Bayesian Ridge.

Bayesian regression techniques include regularization parameters in the estimation procedure; The regularization parameter is not established in a strict sense, but is adjusted to the available data [29]. Bayesian regression estimates a probabilistic model of a regression problem [30]. Application of the Bayes algorithm


$$\exp(J)=\prod_{n=1}^{N}\exp\left(-{\left(y_{n}-\omega^{T}x_{n}\right)}^{2}\right)\exp\left(\lambda \omega^{T}\omega \right)$$ (25)

Bayes App:

To minimize J, we use J/w Therefore,


$$-2X^{T}+2X^{T}X\omega +2\lambda \omega =0$$ (26)

$$\left(X^{T}X+\lambda I\right)\omega =X^{T}Y$$ (27)

$$\omega =\left(X^{T}Y\right)$$ (28)

Because P(w) is Gaussian and close to it, the weights are small.

### 2.3 Evaluation metrics

The evaluation metrics used in this research were:

#### 2.3.1 Precision.

It indicates how well the model can predict the continuous values of the target. Here, we assess how close the predictions are to the actual values.${\hat{y}}_{i}y_{i}$

#### 2.3.2 Mean Absolute Error (MAE).

It represents the average of the variations between the target variable and the projected variables without considering the sign. It does not change significantly if there are extreme values in the information and it is calculated as follows:


$$\frac{1}{n}\sum_{i=1}^{n}\left|y_{i}-{\hat{y}}_{i}\right|$$ (29)

#### 2.3.3 Mean Square Error (MSE).

It determines the average of the squared errors (the discrepancy between the estimated and the estimated), paying special attention to extreme or outliers, and is calculated as follows:


$$\frac{1}{n}\sum_{i=1}^{n}{\left(y_{i}-{\hat{y}}_{i}\right)}^{2}$$ (30)

#### 2.3.4 Average Scaled Absolute Error (MASE).

Assesses the accuracy of a prediction model by comparing the model’s mean absolute error to the mean absolute error of a naïve reference model (such as the value forecast above in time series). This metric allows you to compare the performance of models over different datasets or scale units, as it is dimensionless. A MASE value less than 1 indicates that the model outperforms the naïve model, while a value greater than 1 indicates worse performance. It is calculated as:


$$\frac{\frac{1}{n}\sum_{i=1}^{n}|y_{i}-{\hat{y}}_{i}|}{\frac{1}{n-1}\sum_{i=2}^{n}|y_{i}-y_{i-1}|}$$ (31)

#### 2.3.5 Coefficient of determination (R^2^ or R squared).

This assesses the proportion of the target variable’s variance that the model can explain. For its calculation, the correlation between the target variable and the predictions was established as follows:


$$r=r_{xy}=\frac{n\sum x_{i}y_{i}-\sum x_{i}y_{i}}{\sqrt{n\sum x_{i}^{2}-{\left(\sum x_{i}\right)}^{2}}\sqrt{n\sum y_{i}^{2}-{\left(\sum y_{i}\right)}^{2}}}$$ (32)

#### 2.3.6 Adjusted coefficient of determination (R^2^Adjusted).

Indicates whether the model may be overtuned due to its complexity, and is calculated by


$$1-\frac{\left(1-R^{2}\right)(n-1)}{\left(n-k-1\right)}$$ (33)

#### 2.3.7 Training time and testing time.

Training time refers to the period during which the algorithm categorizes new values according to the defined conditions. And test time is the time required by the algorithm to categorize new values according to the defined conditions based on the test data.

### 2.4 Validation metrics

In order for the results obtained to have practical significance, not only was the traditional method of cross-validation used, i.e., separating the data into training data and test data, but also techniques were used that facilitated the evaluation of the proposed models:

#### 2.4.1 Linearity.

If this condition is not met, the regression algorithm will not mathematically capture the trend, which could indicate that the model is biased and will produce erroneous predictions with new data [31].

#### 2.4.2 Normality of error terms.

To prevent confidence intervals from becoming unstable, error terms should follow a normal distribution, i.e., no unusual points should be present in model validation [32,33]. This condition was verified by plotting the histograms of the distribution of the residues.

#### 2.4.3 Correlation.

A heat map was used to avoid erroneous predictions due to the relationship between independent and dependent variables [34].

#### 2.4.4 Autocorrelation.

To prevent error terms from correlating and thus underestimating the estimated standard error, confidence intervals and prediction intervals are extended [35,36]. If the result of this test is between 0 and 2, the autocorrelation is considered positive; however, if it has values between 2 and 4, it is considered negative. To establish this value, the Durbin-Watson technique was used.

#### 2.4.5 Homoscedasticity.

In order for outliers or extreme leverage values not to disproportionately influence model performance, the variance of the error terms should be as constant as possible [37,38]. This parameter was determined by the residual graph which showed a uniform variance.

## 3. Results

The analyzed dataset was composed of 5041 records per variable. Subsequently, a cross-validation process was carried out, through which the data were divided into 80% for training, which is equivalent to 4033 records per variable, and 20% reserved for tests, corresponding to 1009 records per variable. The results of this validation are presented in Table 2.

**Table 2:** Data distribution for cross-validation.

Statistical	Real	Bayesian Ridge	Lasso	Ridge
**count**	1009	1009	1009	1009
**Mean**	1641.04	1641.21	1641.25	1641.22
**Std**	1091.52	1091.23	1091.36	1091.23
**25%**	600.70	600.17	602.13	600.05
**50%**	1572.60	1568.79	1575.86	1568.69
**75%**	2856.80	2859.40	2863.07	2859.44

**Fig 5:**
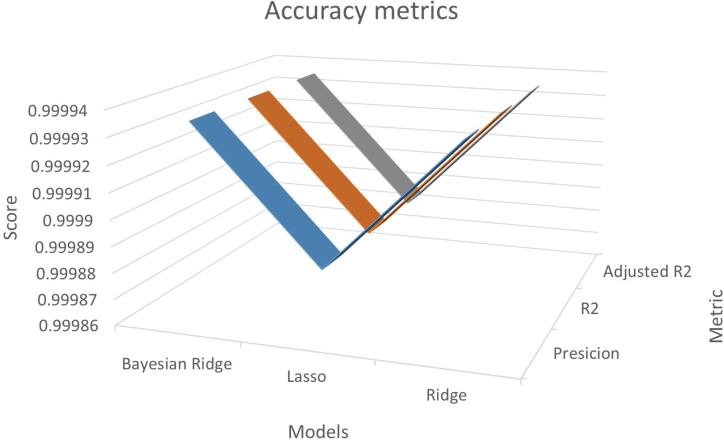
Evaluation metrics: Accuracy.

**Fig 6:**
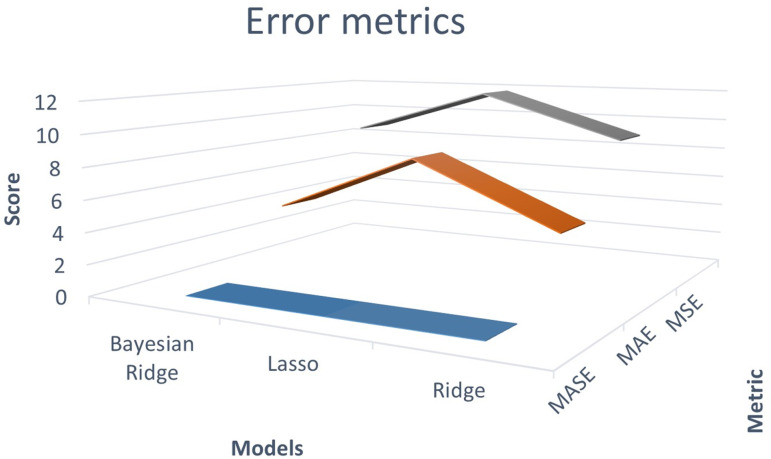
Metrics: Error.

The independent variables selected for the regression model included Alternating Current (AC), AC Voltage, AC Frequency, AC Apparent Power, Direct Current (DC), DC Voltage, and DC Power. Through the application of the Recursive Feature Elimination (RFE) method, it was determined that the AC Reactive Power variable was not statistically relevant and was therefore excluded from the model’s feature set. The results are presented below, accompanied by accuracy metrics in Fig 5, which include accuracy, coefficient of determination (R^2^), and adjusted coefficient of determination (R^2^ adj), as well as error metrics in Fig 6, such as Mean Absolute Error (MAE), Mean Squared Error (MSE), and Mean Absolute Scaled Error (MASE).

The model that offers the highest accuracy is the RFE - Bayesian Ridge, with 0.999935. Similarly, in R^2^, RFE – Bayesian Ridge had the highest value of 0.999935. Similarly, the RFE - Bayesian Ridge has the highest value of 0.999935.

Similarly, for the evaluation metrics: error, we have Fig 6.

Fig 6 shows that the RFE - Bayesian Ridge model presented the lowest values in the evaluated metrics, with a MASE of 0.0034, an MAE of 4.246183 and an MSE of 8.81837. For its part, the RFE - Lasso model recorded a MASE of 0.0065, a MAE of 8.1 and an MSE of 11.7, while the RFE - Ridge model obtained a MASE of 0.0034, a MAE of 4.2 and an MSE of 8.9.

The analysis of the training and testing times of the implemented models revealed significant differences. RFE - Lasso presented a training time of 0.27798 s, which is considerably higher than RFE Bayesian Ridge’s time of 0.003646 s. In contrast, the RFE-Ridge model recorded the shortest training time, with a value of 0.002393 s. In terms of test times, the RFE - Bayesian Ridge model showed a time of 0.001099 s, followed by RFE - Lasso with 0.000994 s. The RFE-Ridge model once again stood out for its efficiency, obtaining the shortest test time with a value of 0.000944 s.

**Fig 7:**
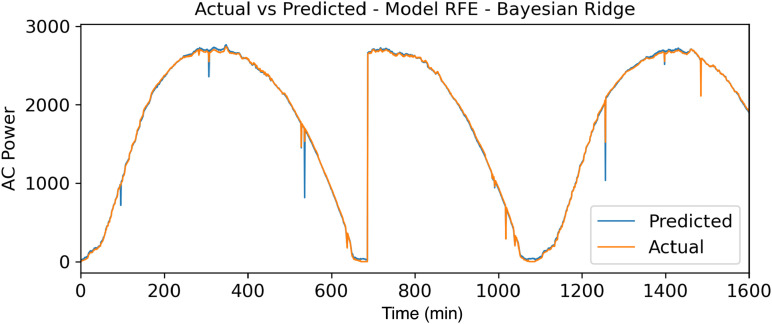
Actuals vs Forecasts – RFE - Bayesian Ridge.

**Fig 8:**
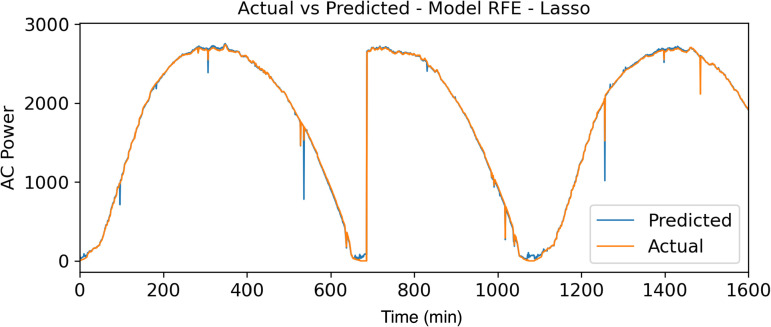
Real Data vs Forecasted Data – RFE - Lasso Model.

**Fig 9:**
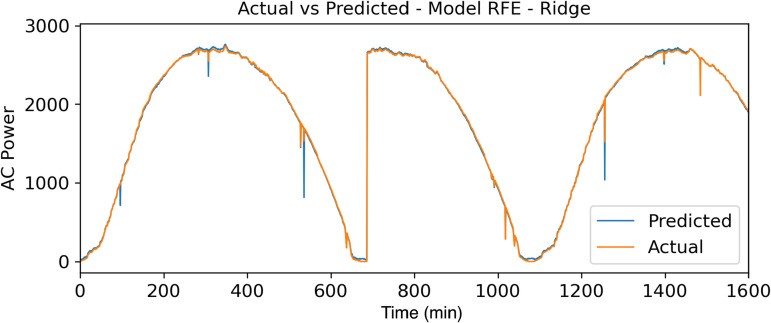
Actuals vs Forecasted Data – RFE – Ridge Model.

To reinforce the results obtained in Figs 7–9, a comparison of the actual data with the forecast data in relation to the power generated (watts) throughout the day (min) can be observed.

### 3.1 Validation metrics

The following results were obtained.

#### 3.1.1 Linearity.

**Fig 10:**
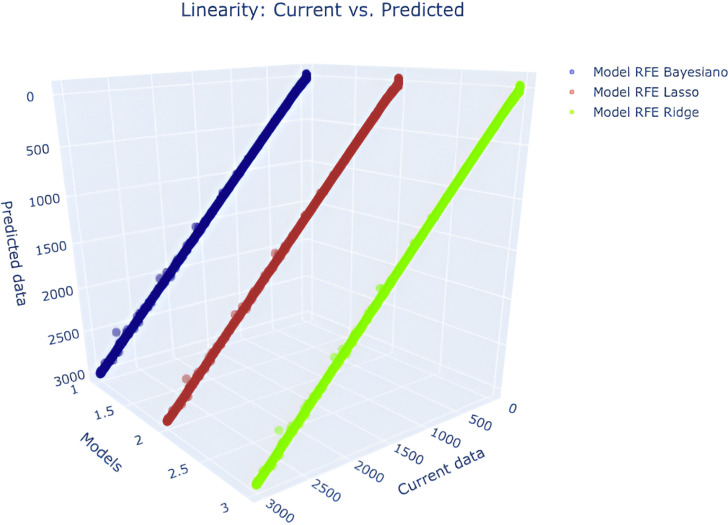
RFE - Bayesian Ridge, RFE - Lasso and RFE - Ridge Linearity: Current vs Predicted.

All three models satisfy the linearity condition, as shown in the scatter plot in Fig 10.

#### 3.1.2 Normality of error terms.

**Fig 11:**
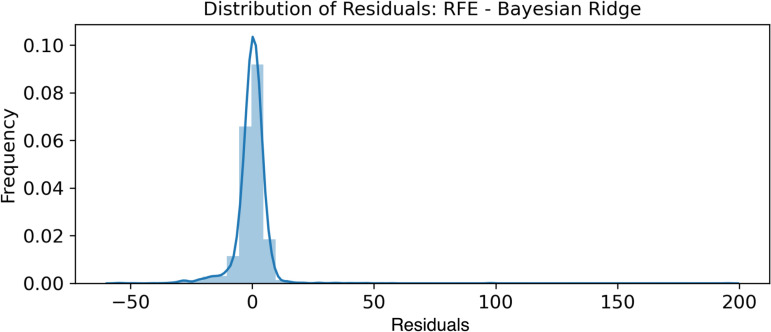
RFE - Bayesian Ridge - Normality of Error Terms.

**Fig 12:**
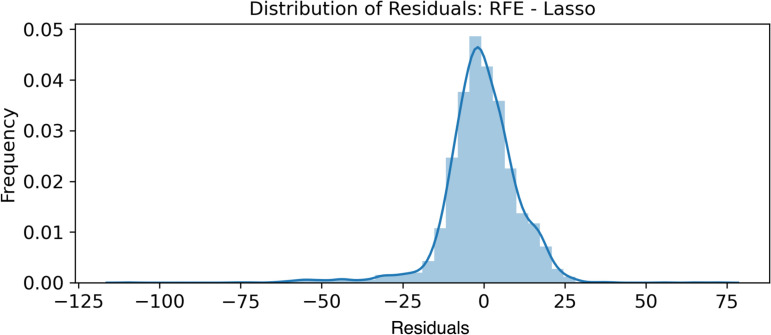
RFE - Lasso - Normality of Error Terms.

**Fig 13:**
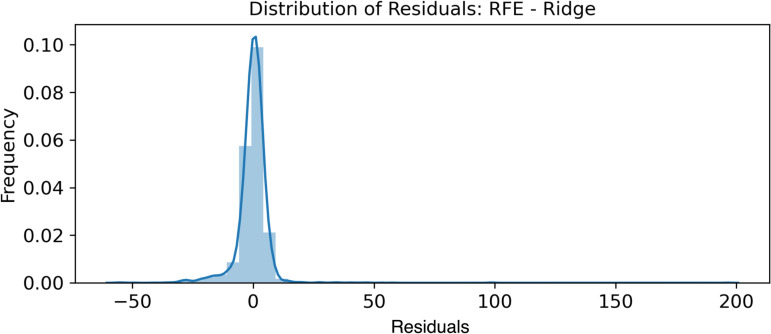
RFE - Ridge - Normality of Error Terms.

The normality of the error terms was verified by plotting the histograms shown in Figs 11–13.

#### 3.1.3 Correlation.

The analysis of the heat maps generated for the three models showed that there was no significant correlation between the independent and dependent variables. This suggests that the relationships between the variables do not introduce biases in the predictions, guaranteeing the absence of errors derived from unwanted correlations in the developed models.

#### 3.1.4 Autocorrelation.

For the three proposed models, the value of the test obtained when applying for the Durbin Watson test was approximately 2, indicating that there was no bias in the proposed models; Therefore, all the information was captured.

#### 3.1.5 Homoscedasticity.

**Fig 14:**
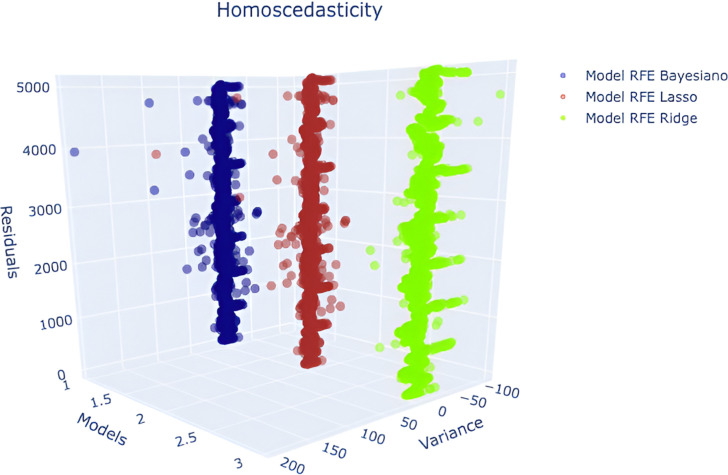
RFE - Bayesian Ridge, RFE - Loop, RFE - Ridge - Homoscedasticity.

For the proposed models, it was determined by the residual graph showing a uniform variance, so there are no extreme values, as shown in Fig 14.

## 4. Discussion

PV systems installed in extreme conditions, such as those located at 3,800 m above sea level, face significant challenges due to environmental fluctuations and climate variability. In this study, three hybrid regression techniques were evaluated to accurately predict power generation in a grid-connected PV system with DC-DC optimizers. The implemented models, RFE-Lasso, RFE-Ridge and RFE-Bayesian Ridge, showed outstanding results, with coefficients of determination (R²) close to 1 (RFE-Bayesian Ridge and RFE-Ridge: 0.99993, RFE - Lasso: 0.99988), minimum values of MAE (RFE-Bayesian Ridge: 4.2461, ridge: 4.2257, RFE - Lasso: 8.099), and equally low mean square error (MSE) (RFE-Bayesian Ridge: 8.8183, RFE - Ridge: 8.8411, RFE - Lasso: 11.7111) In addition, the MASE (Average Scaled Absolute Error) confirmed the robustness of the models, with values of 0.0034 for RFE-Bayesian Ridge and RFE-Ridge, and 0.0065 for RFE-Lasso. These results reflect the superiority of Bayesian and Ridge regularization-based methods in terms of relative accuracy.

Beyond their accuracy, the models stood out for their adaptability to abrupt variations in solar radiation and extreme temperatures, a common challenge at altitudes above 3,800 meters above sea level, as documented in [39,40]. The integration of recursive feature selection (RFE) with Bayesian regularization not only optimized accuracy —as observed in [41], with an R² of 99.99%—, but also improved interpretability by reducing dimensionality, a key factor for its practical implementation [5]. In comparison, approaches such as Random Forest and XGBoost, used in [42–44], achieved an R² of 0.943, but with greater computational complexity.

While the literature on regularization, such as [45,46], focuses on controlled environments, this work extends these techniques to high-altitude conditions, where low atmospheric pressure and intense ultraviolet radiation affect the performance of photovoltaic systems [47]. Recent studies, such as [48], have shown that combining feature selection with assembly methods (e.g., XGBoost) reduces errors in harsh environments, which supports our findings with ElasticNet (30.15% reduction in MAE). Likewise [49,50], underscores the importance of hybrid models to capture nonlinear relationships in extreme climates, an aspect that our approach effectively addresses.

In practical terms, the efficiency of RFE-Ridge and RFE-Bayesian Ridge makes them ideal for use in microcontrollers or embedded systems, as explored in [21,51] for remote solar installations. The homoscedasticity and the absence of autocorrelation in our models guarantee stability in long-term predictions, essential for energy planning in high Andean regions [52] or artificial neural networks (ANNs) [53], which, although accurate, demand greater computational capacity and are not optimized for extreme environments.

Other hybrid approaches, such as those in [48,51], employed optimization techniques (e.g., ChOA) and nonlinear autoregressive models (NARX), with excellent performance in specific predictions. However, the models proposed here stand out for their adaptability to extreme conditions, matching or surpassing these approaches in accuracy and computational efficiency. In addition, this research complements studies such as [54–56], which highlight the relevance of integrating predictive management and feature reduction into PV systems, by directly addressing high-altitude challenges using stacking and hybrid regression techniques.

Finally, this study demonstrates the numerical superiority and practical feasibility of the proposed hybrid models, highlighting their efficiency, adaptability and ease of implementation in extreme conditions. Future research could explore the integration of deep learning techniques to capture nonlinear relationships, as suggested [57,58], or synergies with energy storage systems, according to [59].

## 5. Conclusion

Photovoltaic systems are the most widely used systems in cities located above 3,800 meters above sea level in Peru. To compensate for the disadvantages of these systems, such as low efficiency in power conversion and nonlinear voltage-current characteristics, DC-DC converters are used. Most studies are evaluated under controlled conditions or in prototypes or models implemented in cities or laboratories at sea level; therefore, this research was implemented in the city of Juliaca at 3,800 meters above sea level and in real equipment it tries to demonstrate the efficiency of this type of system. To do this, we rely on Machine Learning and regression techniques for the validation and subsequent design of new systems. Three hybrid regression techniques with variable elimination were implemented: RFE-Lasso, RFE-Ridge and RFE-Ridge Bayesian. Accuracy greater than 0.999% was obtained for the three models generated and an exceptionally low MASE (0.0034 for Bayesian Ridge and Ridge, compared to 0.0065 for Lasso). While the proposed models establish a remarkable advance, their extension using deep learning (DL) techniques could capture even more complex nonlinear relationships, especially under abrupt climate fluctuations. For example, the integration of LSTM (Long Short-Term Memory) networks would allow modeling long-term temporal dependencies in PV power series, crucial for multi-hour forecasts in regions with extreme solar variability. In a complementary way, the use of CNN (Convolutional Neural Networks) would facilitate the spatial analysis of multivariate data, such as irradiance maps or thermographic images of panels, identifying degradation or shading patterns that current linear models do not detect
